# Lateralised visual attention is unrelated to language lateralisation, and not influenced by task difficulty – A functional transcranial Doppler study

**DOI:** 10.1016/j.neuropsychologia.2012.01.015

**Published:** 2012-04

**Authors:** Richard E. Rosch, Dorothy V.M. Bishop, Nicholas A. Badcock

**Affiliations:** a Magdalen College, University of Oxford, UK; b Department of Experimental Psychology, University of Oxford, UK

**Keywords:** Cerebral laterality, Difficulty, Functional transcranial Doppler ultrasound (fTCD), Landmark task, Visuospatial attention, Verbal fluency, Word generation task

## Abstract

Historically, most theoretical accounts of hemispheric specialisation have proposed a single underlying factor that leads to left hemisphere language and right hemisphere visuospatial processing in the majority of people. More recently empirical evidence has started to challenge this view, suggesting lateralisation of language and visuospatial attention are independent. However, so far studies did not control for a possible confound, task difficulty. For this study, 20 healthy right-handed volunteers underwent functional laterality assessment using functional transcranial Doppler ultrasound (fTCD). We assessed laterality using both a word generation task and a novel variation of the visuospatial landmark task that can be adjusted along two dimensions of difficulty (temporal and spatial). The visuospatial laterality measures were highly intercorrelated and unaffected by task difficulty. Furthermore, there was no correlation between visuospatial and verbal lateralisation within individuals – neither qualitatively (in direction of lateralisation), nor quantitatively (in laterality index size). These results substantiate a growing body of evidence suggesting multiple independent biases leading to the hemispheric lateralisation of different cognitive domains, thus further questioning previously accepted models of laterality development and evolution.

## Graphical abstract

**Graphical abstract:**

**Figure:**
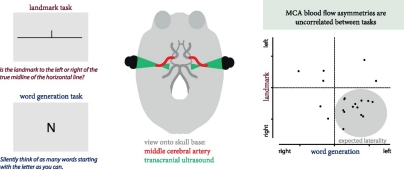

**Highlights:**

► fTCD gives a reliable index of cerebral lateralisation for visuospatial attention. ► Task difficulty has no effect on the laterality index in the visuospatial paradigm. ► Visuospatial and language lateralisation are not correlated within subjects. ► The results challenge single-factor theories of hemispheric specialisation.

## 1 Introduction

Hemispheric specialisation is a prominent feature of cerebral cortical processing. In humans, there is a population bias towards a ‘modal brain’, with functions lateralised to specific hemispheres (e.g., verbal: left hemisphere, visuospatial attention: right hemisphere; Jansen et al., 2004; Knecht et al., 2000; Mesulam, 1999). Various theoretical models posit a single causal factor linking the lateralisation in different cognitive domains (Annett & Alexander, 1996; Cook, 1984), whereby the right hemisphere bias for visuospatial processing is a consequence of language ‘colonising’ the left hemisphere early in development. Recently, however, this view has been challenged by studies that found a lack of correlation between verbal and visuospatial laterality: Whitehouse and Bishop (2009), using functional transcranial Doppler ultrasound (fTCD, cf. Bishop, Badcock, & Holt, 2010; Deppe, Ringelstein, & Knecht, 2004), found no correlation between laterality indices (LIs) from a word generation task and those from a visuospatial memory task. At the population level, there was the usual left-sided bias for the verbal task and right-sided bias for the visuo-spatial task. However, within individuals it was not uncommon to find both functions lateralised to the same side. Similar findings were obtained in a previous fTCD study showing lateralisation of visuospatial attention and language functions to the same hemisphere without functional deficit (Flöel et al., 2001), as well as a functional magnetic resonance imaging (fMRI) study by Badzakova-Trajkov, Haberling, Roberts, and Corballis (2010). The independent lateralisation of different cognitive domains is further supported in functional anatomy studies by evidence for differentially lateralised networks on resting state fMRI (Liu, Stufflebeam, Sepulcre, Hedden, & Buckner, 2009).

One factor that none of these studies considered was the potential effect of varying task difficulties between visuospatial and linguistic tasks. Although there is no agreement as to the nature of the effect, there is evidence that task difficulty can influence cerebral lateralisation. One study comparing easier vs. more difficult cognitive tasks found that increased difficulty led to an increase in laterality (Bodke et al., 2005), but others reported a shift towards more bilateral activation (Helton et al., 2010; Yang, Edens, Simpson, & Krawczyk, 2009). Specifically for laterality assessed by fTCD, increasing task difficulty has been found to lead to more bilateral cerebral perfusion on a standard motor, but not on a word generation task (Dräger & Knecht, 2002), whilst Lust, Greuze, Groothuis, and Bouma (2011) describe different effects of functional lateralisation on single- and dual-task performance.

The effects of task difficulty are also relevant for an understanding of the evolutionary background of laterality: more difficult tasks conceivably require more computational accuracy and neuronal resources; thus both neural processing efficiency (Rogers, Zucca, & Vallortigara, 2004) and neural capacity limitations (Braun, 2007) have been proposed as potential selection pressures contributing to the evolution of lateralisation of cognitive functions, potentially explaining hemispheric specialisation as an adaptation to higher cognitive demands. If the use of unilateral networks was adaptive in this way, one could expect a positive correlation between task difficulty and functional laterality. Whilst for linguistic tasks, no such effect can be observed (Dräger & Knecht, 2002; Dräger et al., 2004), there is little evidence for or against such correlation in visuospatial attentional tasks.

It can be difficult to match task difficulty across different cognitive domains, but by varying difficulty level of a task within a domain, one can clarify how far task difficulty affects lateralisation of different functions within individuals.

Visuospatial processing tasks are generally lateralised, but often show more bilateral activation than verbal processing ones (Clements et al., 2006). Additionally, the difficulty of a classic visuospatial task (bisection/landmark task: Fink et al., 2000; Flöel et al., 2002) can be manipulated easily. A visuospatial task therefore has the potential to be sensitive to intra-individual changes in the degree of laterality depending on differences in task demands. In this study, we present a modified landmark task designed to be variable in difficulty. Laterality measurements from this task were compared to those obtained using a standard word generation (letter-initial verbal fluency) task (Knecht et al., 1998), where in each trial subjects are asked to generate as many words as possible beginning with the letter presented on screen.

## 2 Materials and methods

### 2.1 Subjects

Participants (12 females, 8 males; aged 20–33, median 22) were recruited from Oxford University students and residents in Oxford. Handedness was assessed using the Edinburgh Handedness Inventory (Oldfield, 1971), all subjects were found to be strongly right handed. Only participants that had either full or fully corrected vision were included.

### 2.2 Apparatus

Bilateral blood flow was measured simultaneously using a commercially available Doppler ultrasonography device (DWL Multidop T2: manufacturer, DWL Elektronische Systeme, Singen, Germany), using two 2-MHz transducer probes mounted on a flexible headset. The experiment used Cogent 2000 and Cogent Graphics (www.vislab.ucl.ac.uk/cogent.php) for experimental presentation and stimulus design. Visual stimuli (letters, pictures) were presented on a standard CRT monitor (21 in., *Digital* VRC2143) using Matlab (Mathworks, Natick, MA, USA), which sent parallel-port marker pulses to the Multidop system to mark the start of each epoch. In the landmark task, participants’ responses were recorded through a standard wireless computer keyboard held on their lap.

### 2.3 Stimuli

The word generation paradigm was presented as described in Knecht et al. (1998): A total of 23 trials (one for each letter of the alphabet in random order, excluding the letters Q, X and Z) were presented with the duration of 1 min for each trial. Trials consisted of an initial cueing tone, 5 s during which the words ‘clear mind’ were displayed on the screen, 2.5 s with the letter displayed on the screen and a further 12.5 s with a blank screen during which the participant silently generated words starting with the letter displayed, 5 s during which the participant spoke the generated words, and finally 35 s during which the word ‘relax’ was displayed on the screen.

The landmark paradigm was based on that used by Flöel et al. (2002). Stimuli were presented on the centre of a 21 in. 4:3 computer screen. On each trial, the participant saw a thin horizontal line (visual angle = 5.43°) bisected by a vertical line (visual angle = 0.61°) either to the left or right of the exact middle, followed by a dynamic visual mask (Knibb, 1992). Visual masks consisted of 100 randomly generated lines (50 white, 50 black so as to avoid a uniform black square that the stimulus could integrate with and remain visible as an after-image) spanning an area of 8.14° × 3.26°; novel patterns were created for each mask. On each trial, participants were requested to report the perceived location of the bisecting ‘landmark’ (i.e., left or right of true midline) by pressing one of two buttons on a standard wireless computer keyboard on their lap.

Within a single epoch, participants made six landmark estimates. Thus each epoch consisted of a cueing tone, 5 s during which the words ‘clear mind’ were displayed on the screen, 1 s during which a circular fixation was displayed, six successive trials (randomised order of three bisected left of true midline, three bisected right of true midline) presented at regular intervals of 1700 ms, regardless of response. If a response was made before the next landmark estimate, the response was recorded and participants received visual feedback to acknowledge response recording (i.e., *not* performance feedback) by the presentation of a new line mask. After this activation phase, the screen displayed ‘relax’ for 30 s.

**Fig. 1:**
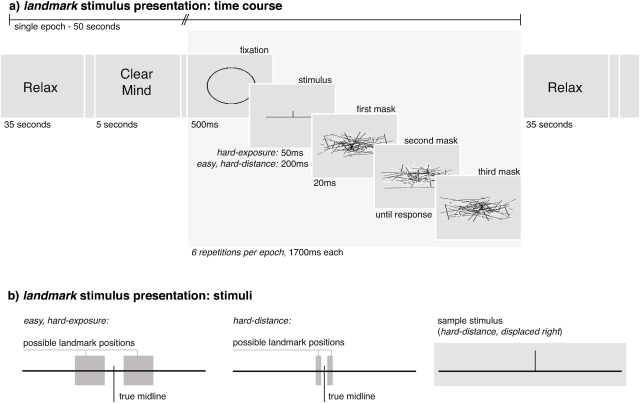
(a) Single epoch in landmark paradigm for the conditions with longer exposure (*easy* and *hard distance*) and shorter exposure (*hard exposure*). (b) A single landmark paradigm stimulus. Shaded areas indicate possible positions for the landmark in the conditions with the landmark far from midline (*easy* and *hard exposure*) and the close to the midline condition (*hard distance*).

The landmark paradigm was run in three conditions that differed in exposure time and distance of the landmark from the true midline – the easy paradigm (landmark far from midline, long stimulus display), the hard-distance paradigm (landmark close to the midline) and the hard-exposure paradigm (short stimulus display) (Fig. 1). For each condition 10 epochs were run – the 30 epochs of the landmark paradigm presented in an individually randomised order for each participant. After epoch 10 and epoch 20 participants were offered a break and given feedback on their performance (i.e., percentage of correct responses and average reaction time) on the screen. The median reaction time and percentage of correct answers were recorded for each epoch as measures of difficulty for the respective task.

### 2.4 Data analysis

The fTCD data were analysed using a custom program based upon Average (Deppe, Knecht, Henningsen, & Ringelstein, 1997). This included down-sampling the data from 100 to 25 Hz, left and right channel normalisation to mean values of 100, heart cycle integration, and artefact rejection. The raw data were trimmed based upon task-specific epochs and normalised on an epoch-by-epoch basis. This normalisation technique involves setting the mean left and right channel activation to 100 within each epoch whereas the traditional approach performs the normalisation across all available data. The technique is useful for removing biases that arise from gradual changes in the Doppler signal across the experimental session.

Epochs including normalised values outside 60–140 were excluded as measurement artefacts: Across all conditions of the landmark paradigm an average of 0.85 (range 0–10) epochs per subject were excluded. For the different conditions of the landmark paradigm, the mean number of exluded epochs per subject are: 0.30 (range 0–3) for easy, 0.20 (range 0–2) for hard-exposure and 0.35 (0–5) for hard-distance. In the word generation paradigm an average of 0.6 (range 0–10) epochs per subject were excluded. Although this resulted in fewer usable epochs, the resulting data were overall less noisy with this exclusion criteria imposed. For each task, baseline-corrected, left minus right difference values were used to calculate LIs.

Individual LIs were obtained by calculating the average left-right difference across a 2-s window centred on the maximum peak difference within a task-specific period of interest (POI) for all accepted epochs. Positive values indicate left lateralisation and negative values, right lateralisation. Task-specific baseline and POI values (in seconds) were used relative to the initial stimulus event markers; word generation paradigm: baseline = −13 to −3, POI = 8–18; landmark paradigm: baseline = −15 to −5, POI = 10–20. The internal consistency of LI measures for individuals was assessed calculating Cronbach-*α* based on independently calculated LIs for each trial as well as calculating the Pearson's correlation coefficients for the condition pairs.

Statistical analysis was conducted using several different tests: for comparisons over not normally distributed data (i.e. behavioural measure of number of correct responses) we used the non-parametric Friedman test with the non-parametric post hoc Dunn test. To compare mean reaction times and LIs across the three landmark conditions, univariate repeated measures ANOVAs were conducted with Bonferroni post hoc testing, if the ANOVA was significant. For comparison of mean LIs between the two main paradigms, a *t*-test was used. Additionally, a linear regression of the correlation between word generation laterality and landmark laterality within individuals was conducted. Results with *p* < 0.05 were accepted as statistically significant.

## 3 Results

### 3.1 Behavioural difficulty measures

**Fig. 2:**
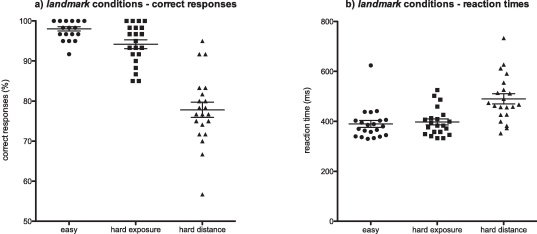
(a) Percentage of correct (left vs. right) landmark estimates in the three different conditions. (b) Reaction time for landmark estimates in the three different conditions. Individual points each indicate the average for a subject across all epochs of that condition.

Behavioural measures of difficulty for the landmark paradigm (mean percentage of correct responses, mean reaction time) differed significantly between the three landmark conditions. Using the non-parametric Friedman test, the percentage of correct responses (which showed skewed distributions due to ceiling effect) were significantly different between conditions at *p* < 0.0001, Friedman *χ*^2^(2) = 36.22; subjects responded correctly most often in the easy condition (98.0%), followed by the hard-exposure (94.2%) and hard-distance (77.8%) conditions. On univariate repeated measures ANOVA, reaction time also differed between conditions; *F*(2, 62) = 40.72, *p* < 0.0001, *R*^2^ = 0.67, with reaction times being shortest for the easy (389.7 ms), followed by the hard-exposure (397.8 ms) and then hard-distance (490.1 ms) conditions (Fig. 2).

Post hoc testing confirmed significant differences between *hard-distance* and both others, but there were no significant difference between the *easy* and *hard-exposure* conditions in percentage of correct answers or reaction time. Non-parametric post hoc Dunn test for percentage of correct answers; *easy* vs. *hard-exposure*, rank sum difference = 12.0, *p* ≥ 0.05; *easy* vs. *hard-distance*, rank sum difference = 37.5, *p* < 0.05; *hard-exposure* vs. *hard-distance*, rank sum difference = 25.5, *p* < 0.05. Bonferroni post hoc test for reaction time: *easy* vs. *hard-exposure*, *t* = 0.65, *p* ≥ 0.05, *d* = 0.29; *easy* vs. *hard-distance*, *t* = 8.12, *p* < 0.05, *d* = 2.06; *hard-exposure* vs. *hard-distance*, *t* = 7.45, *p* < 0.05, *d* = 1.90 (Fig. 2).

### 3.2 Laterality indices

**Fig. 3:**
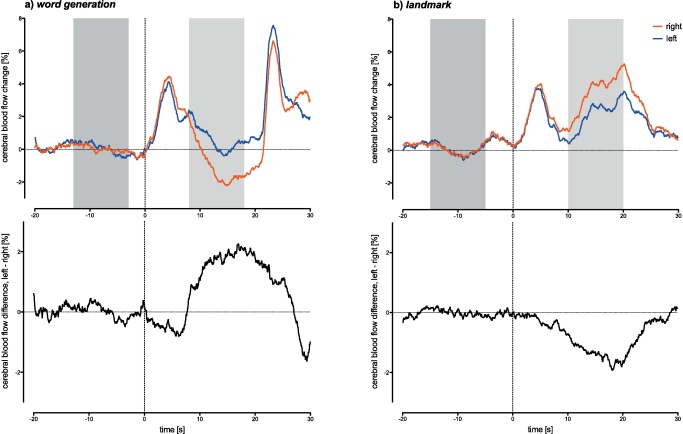
Functional Doppler activation plots for (a) the word generation and (b) the combined landmark paradigm. The top part of the diagram indicates the blood flow in each of the two Doppler channels (red: right channel, blue: left channel); the bottom part shows the difference between the two channels over time. (For interpretation of the references to colour in this figure legend, the reader is referred to the key in the top right hand corner or the web version of the article.)

Mean activation during the periods of interest averaged across all respective epochs did not differ between the different landmark conditions on repeated measures univariate ANOVA: *F*(2, 59) = 2.85, *p* = 0.45, *R*^2^ = 0.58 with mean (SD) activations: *easy* = 2.23 (0.63); *hard-distance* = 2.35 (0.76); *hard-exposure* = 2.13 (0.71). Activation averaged across all landmark epochs vs. word generation epochs differed significantly on a *t*-test: *t*(19) = 3.92, *p* < 0.001 with mean (SD) activations: landmark = 2.24 (0.54); word generation = 0.85 (3.88). This reflects that for several of the word generation epochs, activation during the trial phase would dip below baseline (cp. Fig. 3).

Mean LIs did not differ significantly between the individual landmark conditions (*easy*, *hard-distance*, *hard-exposure*) on a repeated measures ANOVA: *F*(2, 59) = 0.58, *p* = 0.56, *R*^2^ = 0.03. Mean (SD) LIs were: *easy* = −1.94 (3.33); *hard-distance* = −2.38 (2.9), *hard-exposure* = −1.66 (3.83); the three different landmark conditions showed a high internal consistency within individuals (Cronbach-*α* = 0.819). Furthermore, the data sets were all significantly correlated, with Pearson correlation coefficients (*p*-value) of: *easy* vs. *hard-distance* = 0.53 (0.011); *easy* vs. *hard-exposure* = 0.61 (0.004); *hard-distance* vs. *hard-exposure* = 0.67 (0.001). Hence for the following analysis the landmark conditions are averaged together when compared to the word generation paradigm. Group LIs for the combined landmark paradigm and the word generation paradigm differed significantly (*t*-test, *t* = 3.76, *p* = 0.0013, CI_95_: −5.81 to −1.66) with a mean difference of −3.74 between landmark and word generation paradigm, indicating a low/negative LI for the landmark paradigm (right hemispheric blood flow > left hemispheric blood flow) and a high/positive LI for the word generation paradigm (right hemispheric blood flow < left hemispheric blood flow). Left and right blood flow velocities for the word generation and the landmark paradigms, averaged across all subjects and epochs within the respective paradigm are shown in Fig. 3, showing the time course of the observed blood flow changes and the temporal dynamics of their hemispheric difference.

### 3.3 Cross-domain laterality correlation

**Fig. 4:**
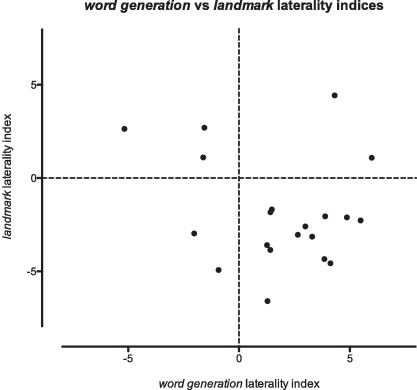
Correlation between laterality indices (LIs) within individuals. Individual points indicate word generation and landmark laterality indices for a single participant.

There was no significant correlation between word generation and landmark LI within individuals; the linear regression did not differ significantly from zero at *r* = 0.19, slope: −0.19, CI_95_ −0.67 to 0.29; *p* = 0.42 (Fig. 4).

## 4 Discussion

The novel landmark paradigm introduced in this study (modified from Flöel et al., 2002) proved successful in eliciting group LIs consistent with the current literature on visuospatial attention lateralisation (e.g. Flöel et al., 2002; Jansen et al., 2004; Mesulam, 1999). The difficulty manipulation had a clear effect on behaviour, as evidenced by the behavioural data on percentage of correct responses and reaction time. However, task difficulty had no effect on the LI. Furthermore, both the overall activation and LIs for the landmark and the word generation paradigms were of comparable magnitude.

In this group of healthy young right-handers, there is a clear majority in left-*word generation*/right-*landmark* lateralised individuals, which is to be expected from previous studies assessing the prevalence of cerebral lateralisation in relation to handedness (Clements et al., 2006; Knecht et al., 2000). However, there was no correlation between strength and direction of word generation and landmark lateralisation within individuals, and participants with atypical laterality (right-*word generation* or left-*landmark*), were present in all possible permutations (i.e., in all four quadrants of the diagram in Fig. 4). These findings substantiate previous reports that showed the possibility of lateralisation of verbal and visuospatial functions to the same hemisphere without functional impairment on fTCD assessment (Flöel et al., 2001; Lust et al., 2011; Whitehouse & Bishop, 2009). This evidence also ties in with twin studies that have previously highlighted differentially laterality discordant twins, suggestive of a developmental spread of lateralising influences (e.g. Gurd, Schulz, Cherkas, & Ebers, 2006; Lux et al., 2008; Rosch, Ronan, Cherkas, & Gurd, 2010; Yoon, Fahim, Perusse, & Evans, 2010). Such findings are inconsistent with models of laterality relying on a single factor, such as the right shift hypothesis, or early hemispheric injury as the main developmental driving factor for atypical laterality (Clark, Klonoff, & Tyhurst, 1986) – models with a single factor would predict a correlation between the different measures of laterality.

On the contrary, this evidence is in favour of (at least) two independent developmental factors influencing mature cerebral laterality. If independent factors influence the two measures of laterality described, each with a preferential influence towards typical lateralisation (visuospatial: right, verbal: left), the predicted outcome would match the observation of this study: with the majority of subjects being typically lateralised, but no cognitive deficit in those that show any degree of atypical laterality, including the two assessed functions lateralising to the same side.

Understanding the independent factors at play has potentially significant clinical relevance: in assessing and potentially treating the underlying developmental processes leading to functional deficits associated with classically lateralised cognitive function (such as specific language impairment and dyslexia) as well as in predicting and treating the cognitive sequalae of hemispheric injuries at different stages in hemispheric specialisation. This could extend into rehabilitation after later life insults, too: There is resting state fMRI evidence that functional lateralisation is influenced by independent factors not only across cognitive domains, but also functional anatomical networks (Liu et al., 2009). Seeing that functional lateralisation in adults has been found to predict susceptibility to unilateral brain lesions (Knecht et al., 2002), understanding the exact functionally lateralised anatomy of the adult brain can help identify those patients most likely to benefit from targeted interventions after localised injuries such as strokes.

On an evolutionary scale, these results contradict potential constraints commonly presented as selection factors for the evolution of hemispheric lateralisation in humans, such as the argument that neural capacity limitation requires a division of labour between the hemispheres (Braun, 2007). Both existing clinical evidence of early life hemispherectomies and their astonishing potential outcomes (Vargha-Khadem & Polkey, 1992) and the evidence presented here are inconsistent with limited neuronal capacity within a hemisphere being a significant limiting factor to cognitive function.

Although in studies of dual task performance, an interaction between functional lateralisation and performance could be observed, there was no quantitative correlation between LIs and performance (Lust et al., 2011). If neuronal capacity limitations within a hemisphere were causative in directing an individual's functional lateralisation, the laterality of different functions would be associated in that they would preferentially ‘divide’ the hemispheric resources amongst themselves. In those cases where they do not, one would then predict performance limitations – neither of these effects has been observed in this study.

Our understanding of how and why the human brain lateralises functions has been changing over the last decade, due to the increased availability of empirical data to test hypotheses that have been discussed for decades. Our study alongside many in the current literature shows that hemispheric specialisation is not a unidirectional process driven by a single factor, but instead should be reconsidered for different cognitive domains and anatomical networks. The further study of the developing brain, for which functional transcranial Doppler as used here is particularly appropriate, is likely to allow for the testing of new, domain specific models of hemispheric specialisation and should be the next step in the field.
